# Hypofertility in a persistence of mullerian duct syndrome: Case report

**DOI:** 10.1016/j.ijscr.2020.11.011

**Published:** 2020-11-27

**Authors:** Issam Jandou, Tarik Mhanna, Mehdi Chennoufi, Mohamed Aynaou, Amine El houmaidi, Ali Barki

**Affiliations:** a Mohamed VI University Hospital, Oujda, Morocco; b Faculty of Medicine and Pharmacy, Mohamed Ist University, Oujda, Morocco; c University Hospital Center Ibn Rochd, Casablanca, Morocco; d Faculty of Medicine of Casablanca, Morocco

**Keywords:** PMDS, syndrome of persistent Müllerian ducts, AMH, anti-Mullerian hormone, CT, computerized axial tomography, ELISA, enzyme-linked immunosorbent assay, Mullerian duct syndrome, Anti-Mullerian hormone, Transverse ectopic testis, Hypofertility, Disorders of sexual development

## Abstract

**Introduction:**

Persistent Mullerian duct syndrome is a rare form of pseudohermaphroditism, possibly resulting from the absence of Mullerian duct inhibiting factor.

**Presentation of case:**

We report the case of a 36 year-old patient with a syndrome of persistent Müllerian ducts (PMDS) of the female type. The Clinical examination revealed avacant scrotum, a normal penis. The spermogram analysis found azoospermia. Imaging using ultrasound and tomotensidometry found the presence of amass in the back of bladder.

A Laparoscopic exploration revealed two slightly enlarged testicles, in a pelvic situation intimately confined to a rudimentary uterus with two tubes. The histological examination was in favor of a uterine endometrium and two fallopian tubes. The karyotype requested later was male 46 XY.

**Discussion:**

Since the first case, between 200 and 262 cases have been reported in literature around the world, this might reveal that the incidence and prevalence of this type of diseases are not well-estimated. The diagnosis is often a fortuitous discovery during a cryptorchidism surgery or inguinal hernia, more rarely during the management of a state of infertility or the degenerescence of the testis or Mullerian derivative. It is caused by either the anti-Mullerian hormone deficiency (AMH) or a dysfunction of its receptor.

**Conclusion:**

A multidisciplinary approach is necessary for surgical management. long-term prognosis is favorable, though fertility seem to be decreased and also it might lead to a riskiness of malignancy owing to cryptorchidism and preserved Mullerian remants.

## Highlights

• PMDS is a rare, autosomal recessive sexual development abnormality characterized by the coexistence of Mullerian derivatives in a male phenotype and genotype patient.
• Can be identified during the surgery of cryptorchidism, and more rarely by an ectopic testicular degeneration.
• The PMDS has a good prognosis despite the increased risk of infertility as well as testicular and Mullerian degeneration.
• The treatment requires a multidisciplinary approach.

## 1 Background

Muller's mesonephritic channel persistence syndrome is a very rare form of abnormality of sexual development characterized by the presence of Mullerian structres (upper vagina, uterus, fallopian tubes) in subjects of male genotype and phenotype [1].

The diagnosis is often a fortuitous discovery during a cryptorchidism surgery or inguinal hernia, more rarely during the management of a state of infertility or the degenerescence of the testis or Mullerian derivative. It is caused by either the anti-Mullerian hormone deficiency (AMH) or a dysfunction of its receptor [2,3].

PMDS is a rare form of internal pseudo-hermaphroditism, since the first case, between 200 and 262 cases have been reported in literature around the world [4,5]. We report the case of a 36 year-old patient with a syndrome of persistent Müllerian ducts (PMDS) of the female type. The work has been reported in line with the SCARE criteria [6].

## 2 Case presentation

The described case was a late diagnosis of a 36-year-old patient, who was examined for primary infertility after 2 years of marriage. In terms of physical appearance, the patient was 1.65 m tall, thin, with normal pubic and axillary hair. Examination of the external genitalia revealed a normal size of the penis and an empty scrotum. Other physical findings were normal, and the family history was unremarkable.

As part of its infertility, a spermogram revealed total azoospermia and leucospermia. Epididymal markers (alpha 1–4 glucosidase, carnitine) were collapsed while the prostatic markers (citrate, acid phosphatase) and seminal vesicles (fructose) were normal. The hormone balance showed a marked increase in LH and normal levels of FSH and testosteroneemia.

**Fig. 1:**
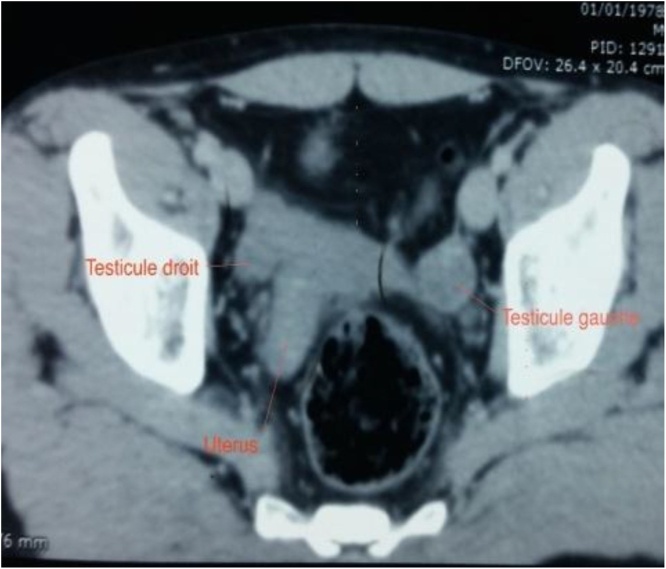
Pelvic CT showing uterus and two intra-abdominal testicles.

We performed an abdominal, pelvic and scrotal ultrasound that revealed an empty scrotum and a hypoechoic pelvic mass. Abdominopelvic CT showed a retrovesical pelvic formation of homogeneous tissue density, with both intrapelvic testes (Fig. 1).

**Fig. 2:**
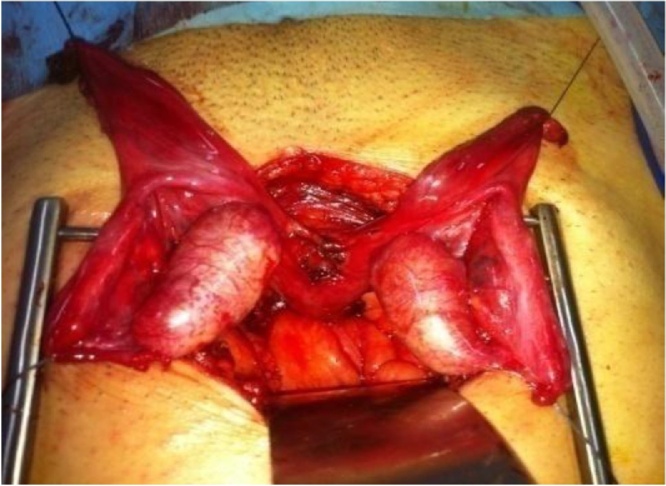
Intra-operative view of the uterus, fallopian tubes, and testes after round ligaments and peritoneal attachments.

**Fig. 3:**
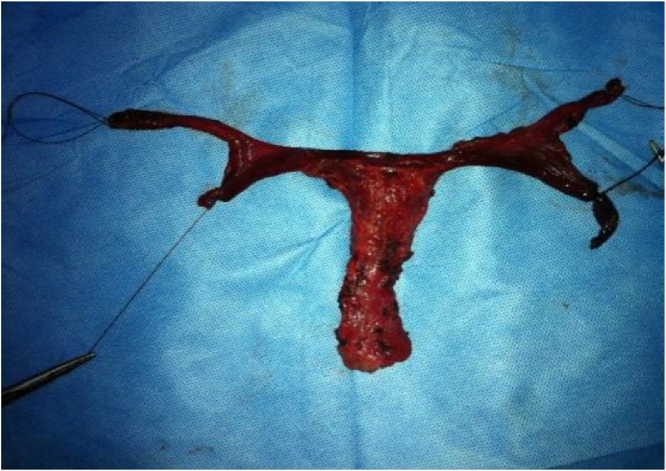
Hysterectomy piece with bilateral adnexectomy.

Laparoscopic exploration showed two slightly enlarged testicles, in a pelvic situation intimately confined to the tissue formation described on the CT scan which corresponds to a rudimentary uterus and two fallopian tubes. It was decided to convert into a medial incision under umbilical (Fig. 2). Both gonadal masses were dissected and released from both tubes. The two different channels dissected to the prostatic base had blind ends. The seminal vesicles were of normal size on either sides of the uterus that was implanted in the prostatic base. A hysterectomy with bilateral adnexectomy was performed (Fig. 3).

The bilateral testicular descending over the two inguinal orifices to the scrotum was not possible due to the insufficient length of the two pedicles. It was decided to externalize both testicles at the superficial orifices of the inguinal ducts to maintain androgenic secretion and to make the two testicles accessible to self-monitoring by palpation.

The anatomopathological result was in favor of a uterine endometrium and two fallopian tubes.

The karyotype requested later was male 46 XY.

The postoperative course was favorable and the patient would eventually benefit from a testicular biopsy with an intracytoplasmic injection of spermatozoa.

## 3 Discussion and conclusion

The first published account of PMDS was described in 1939. Alfred Jost's distinguished studies on undifferentiated gonads of rabbit embryos have highlighted the etiology of PMDS [7]. They found out that sexual differentiation is determined by two testicular hormones of different origin and function. On the one hand, a hormone produced by leugyg cells, masculinizing (testosterone). On the other hand, (anti-Mullerian hormone) secreted by sertoli cells whose role is the inhibition of Mullerian ducts [8,9].

Only 150 cases of PMDS were reported in the literature in 1993, and between 200–260 cases to date [4]. Gradually, more cases have been reported because of the large number of early surgical procedures for cryptorchidism. Ethnic mapping has showed a broad and equitable distribution of PMDS [10]. But, there has been a predominance of AMH mutations in the Arab and Mediterranean populations, and a higher proportion of AMH-RII mutations are observed, especially in the populations of northern Europe and the USA [[10], [11], [12]]. The prevalence of PMDS may be underestimated due to the lack of knowledge in the part of practicing physicians and many cases that are accidentally diagnosed during an imaging or cryptorchid surgery. Transmission is autosomal recessive, which could be either a homozygous or heterozygous composite, with a higher rate of homozygosity in the Arab and Mediterranean countries which is caused by a strong consanguinity. These anomalies affect only male figures, while the females are healthy carriers with normal fertility and development [11].

Despite the fact that the majority of PMDS are linked either with mutations of the AMH gene or its receptors, a small number of patients remain without discovered mutation, which means, in turn, without identifiable etiology. The molecular studies of belville and al on more than 113 families with PMDS showed that 44.2% had a mutation of the AMH gene, 42.5% had a mutation of the AMH-RII gene and more than 13% had an unknown etiology [12].

PMDS is a rare, autosomal recessive sexual development abnormality characterized by the coexistence of Mullerian derivatives in a male phenotype and genotype patient. Until the 8th week of embryonic development, the presence of the two male and female genital tracts coincides: the Muller and wolff channels [13,14]. In humans, the secretions of AMH and testosterone by the fetal testicles are responsible for sexual differentiation in morphotype male. In the normal state alone sessile hydatid, prostatic utricle and verumontanum.

At birth, men who are affected by PMDS have a male phenotype, normally virilized. It can be often identified during the surgery of cryptorchidism, and more rarely by an ectopic testicular degeneration by the discovery of Mullerian structures [1]. The PMDS can be presented in three anatomical forms:

1. In 60–70% of the cases, both testicles are located in the abdomen contiguous to the uterus via the fallopian tubes.
2. In 20–30% of the cases, a testicle is lodged in the scrotum and the uterus while the contralateral testicle is in an inguinal hernia on “uteri-inguinlis hernia”.
3. 10% of the cases show transverse testicular ectopia, in which both testicles are housed in the same hereditary sac attached to the two fallopian tubes.

There is an excessive mobility of the tests in PMDS patients, which increases the risk of torsion in them. Besides, the testicle has a normal structure and produces normal testosterone levels. Other abnormalities may be associated with PMDS, namely aplasia of the epididymis, and a dissociation between the epididymis and the testis [15].

The etiological diagnosis can be guided by the assay of the blood AMH by means of an ELISA test. The AMH primary receptor mutations are characterized by normal AMH levels, but it is very low or even undetectable in the eyes of the patients with mutations of the AMH gene [5]. The synthesis of AMH is inhibited by testosterone only after puberty and becomes undetectable with a preserved virilization [16]. Hypofertility may be a complication of cryptorchidism or a consequence of anatomical abnormalities affecting the excretory ducts [17].

Ultrasound is commonly used for the study of the internal anatomy of patients with sexual ambiguity. According to Mairi Stevenand al, pelvic ultrasound has an interest in a first evaluation of Mullerian structures with low sensitivity 54% and specificity 50% [18]. A comparative study between pelvic MRI and pelvic ultrasound showed a clear superiority of the magnetic resonance imaging that elaborates a good resolution of contrast and images in the different plans. The ultrasonic is a simple, available and reproducible technique [19]. Despite its invasiveness, laparoscopy is the gold standard in the diagnosis of PMDS, it allows excellent investigation and visualization of Mullerian structures and contributes to the correct diagnosis [20,21].

PMDS is usually fortuitous for intra-operative discovery in the treatment of cryptorchidism or inguinal hernia, but rarely for testicular tumor or degeneration of Mullerian structures. The management of this type of anomaly of sexual differentiation poses a dilemma. Once the diagnosis is made, an orchidopexy can be performed to preserve the fertility of the patient and for possible monitoring of the testicle in the inguinal position [22,23]. Anatomically, the different canal describes a parallel path to the lateral wall of the uterus after having left the testicle. The insufficient length of the different canal makes it difficult for orchidopexy. A traumatic dissection is not feasible. Only the salpingectomy and hysterectomy with careful dissection of the the vasa deferantia allows orchidopexy under a slight tension [24]. Or the division of the uterus in the median line to allow translocation of both testicles, each into the corresponding scrotum through the inguinal canal with possible destruction of the uterine endometrium to prevent further degeneration [25].

Moreover, the preservation of the uterus and the upper 1/3 of the vagina can be complicated by urinary infection [24]. If the PMDS is associated with testicular malignant transformation, the management will be similar to other testicular tumors [25].

An internal pseudo-hermaphroditism is characterized by a male genotype with a 46 XY and a male phenotype with normal secondary sexual characteristics, in which one notes a persistence of Mullerian ducts.

PMDS is most often associated with unilateral or bilateral cryptorchidism, incidental discovery during an intervention for testicular ectopia or inguinal hernia. It is an abnormality that responds to a recessive autosomal genetic transmission in most cases due to a mutation of either the type II receptor gene of AMH or a mutation of the AMH gene it self, which results in a difference in AMH levels in PMDS patients.

Several cases of paternity were described worldwide. The PMDS has a good prognosis despite the increased risk of infertility as well as testicular and mullerian degeneration. For this reason, the treatment this disease requires a multidisciplinary approach, a surgeon, an endocrionologist, a radiologist and a geneticist.

## Declaration of Competing Interest

The authors report no declarations of interest.

## Funding

We have any funding for your research.

## Ethical approval

The consent to publish this information was obtained from study participants. We confirm that **written** proof of consent to publish study participants are available when requested and at any time.

## Consent

The consent to publish this information was obtained from study participants. We confirm that **written** proof of consent to publish study participants are available when requested and at any time.

## Author’s contribution

Dr. IJ, Dr. AT, Dr. YL Dr. WB analysed and performed the literature research, Pr. MD, Pr. AD, Pr. RA performed the examination and performed the scientific validation of the manuscript. Issam Jandou was the major contributors to the writing of the manuscript. All authors read and approved the manuscript.

## Registration of research studies

Not applicable.

## Guarantor

Issam Jandou.

## Provenance and peer review

Not commissioned, externally peer-reviewed.
